# Nationalism in New Zealand Media During the COVID-19 Pandemic: A Mixed Methods Study

**DOI:** 10.1093/phe/phaf009

**Published:** 2025-09-05

**Authors:** Emma M R Anderson, Elizabeth Fenton, John A Crump

**Affiliations:** Bioethics Centre, Division of Health Sciences, Dunedin School of Medicine, University of Otago, Dunedin, New Zealand; Bioethics Centre, Division of Health Sciences, Dunedin School of Medicine, University of Otago, Dunedin, New Zealand; Centre for International Health, University of Otago, Dunedin, New Zealand

## Abstract

The COVID-19 pandemic has revealed the complex interplay between national self-interest and global cooperation. Media communication can contribute to the formation of national identity and promote nationalist themes, particularly in times of crisis. Media portrayals of the nation during a pandemic are informative, since nationalism, specifically health nationalism, may undermine the popular appetite for and effectiveness of global response efforts. We sought to investigate whether nationalist sentiment was present in COVID-19 reporting in New Zealand media. Using qualitative and quantitative thematic analysis, we identified nationalist themes in the New Zealand media during the COVID-19 pandemic and observed how they changed over the course of the pandemic. We randomly selected 1300 articles from 19 New Zealand newspapers from January 2020 to June 2022. We identified four nationalist themes in New Zealand media reporting during the pandemic: domestication, unification, securitization, and separation. The emergence of nationalism in New Zealand media during the COVID-19 pandemic is an important consideration when developing public health policy. While nationalist sentiments in New Zealand media during the COVID-19 pandemic provided a foundation for domestic solidarity, we argue that balancing these approaches with cosmopolitan appeals to collective humanity would support policies and responses that address both local needs and global inequities.

## Introduction

The coronavirus disease (COVID-19) pandemic, caused by severe acute respiratory syndrome coronavirus 2 (SARS-CoV-2), revealed the complex interplay between national self-interest and global cooperation (Bieber, 2022). Phrases like ‘coronationalism’ (Ozkirimli, 2020) and the ‘pandemic of nationalism’ (Nossem, 2020) highlighted the extent to which many governments adopted a domestic rather than global focus in response to the pandemic (Nossem, 2020). This tendency creates both moral and practical barriers to achieving an equitable global health response.

Nationalist perspectives contend that justice is most effectively realized within the confines of the nation-state, where governments have direct accountability to their citizens. On this view, obligations to nonnationals are often framed more as acts of charity than as duties of justice, thereby permitting states to prioritize their own populations in times of crisis (Rawls, 1993; Miller, 1995; Tamir, 2015). Nationalism is often justified on instrumental grounds as a means to foster social solidarity and trust, which can be valuable for mobilizing domestic responses, particularly to a public health crisis (West-Oram, 2021). Under such conditions, nationalism can harness national identity to support global cooperation (Miller, 2013; de Campos, 2017). However, global solidarity that depends on self-interest is not guaranteed, and the charity framework of cooperation associated with nationalist approaches can be reactive rather than addressing long-term, sustainable, and justice-oriented system strengthening at a global level (Lencucha, 2013). The prioritization of the interests and well-being of citizens and residents over nonnationals can be used to justify an exclusionary approach to resource allocation and policymaking. For example, in the context of global and public health emergencies, the inward-looking orientation of nationalism risks entrenching global health disparities as it permits policies such as vaccine hoarding, export restrictions, and border closures that, while beneficial for domestic populations in the short-term, ultimately undermine global cooperation and exacerbate inequities (Bieber, 2022; Crump et al., 2023).

By contrast, a cosmopolitan perspective challenges the notion that national membership or citizenship determines the scope of moral and political obligations (Ruger, 2009; Brock and Hassoun, 2013; Johnson et al., 2023). On this view, health justice should be grounded in universal principles that are unconstrained by geopolitical boundaries. Indeed, the United Nations Declaration of Human Rights, a paradigmatically cosmopolitan document, declares the individual as the unit of moral worth, not the state to which they belong (Lencucha, 2013). Relevant to global health, a strength of the cosmopolitan view is its recognition of the requirement for international cooperation to respond effectively to infectious diseases that do not respect national borders (Lencucha, 2013). Cosmopolitanism is not only an ethical imperative but also a pragmatic necessity in an interconnected world as protecting the health of others ultimately contributes to the health security of all (Jecker et al., 2022; Kataria and Qu, 2022).

Even for states acting in self-interest, global health security cannot be achieved in isolation. New Zealand’s success in eliminating COVID-19, though widely praised, highlights this tension. While nationalist sentiments helped generate domestic solidarity (Jenne, 2022), these sentiments also contributed to policies that prioritized national success over global health equity (Crump et al., 2023). Nationalist attitudes in New Zealand are not surprising, as empirical analyses of survey data have documented high levels of nationalism in the country (de las Casas, 2008; Skilling, 2010). However, by embracing a global health ethic rooted in cosmopolitan principles, New Zealand and other states can move beyond short-term, insular policies and work toward sustainable, equitable health solutions that recognize a commitment to addressing the shared vulnerabilities and interdependencies of all people, regardless of where they live.

While much of the discussion on nationalism and cosmopolitanism during the pandemic has been theoretical or policy-oriented (Anderson, 1983; Bieber, 2022), there remains a need for empirical research that examines how these ideas manifest in public discourse. The media have played a central role in shaping public understanding of COVID-19, serving as a key source of information even for those who do not regularly engage with news media (Casero-Ripollés, 2020). As a trusted institution in many countries, the media not only informs but also acts as a platform for public discourse and government accountability (Bahador et al., 2016). However, media coverage is often domesticated, emphasizing national perspectives and reinforcing nationalistic themes (Moran, 2009; Skey, 2022). Beyond its informational role, the media is deeply embedded in national identity formation, shaping collective perceptions through coverage of politics, sports and national events (Schlesinger, 1991; Arnold, 2021). Studies have shown that media narratives often employ rhetorical strategies that reinforce national distinctions, particularly during moments of crisis or celebration, where language such as ‘us’ and ‘we’ fosters a sense of unity and belonging (Billig, 1995; Mihelj et al., 2009).

While globalization might suggest a diminishing role for national identity in media, it has, in some cases, strengthened efforts to construct and maintain national branding for global economic positioning (Skilling, 2010; Skey, 2022). The media’s dual role as both an information source and a reflection of societal values makes it a valuable lens for examining the presence and strength of nationalism in a specific context, as it not only shapes public discourse but also mirrors the ways in which societies construct and reinforce national identity (Billig, 1995; Macnamara, 2005; Skey, 2022).

### Purpose of Study

Analyzing media narratives offers an opportunity to empirically investigate how nationalism was expressed and evolved during the COVID-19 pandemic (Otten, 1992; Skey, 2022). In this study, we sought to identify whether nationalist sentiment was present in COVID-19 reporting in New Zealand during the period January 2020 to June 2022, and if so, whether it changed over time. Other media analyses on nationalism and COVID-19 have narrowed search terms to explore nationalism and sport, or COVID-19 reporting in direct comparison with another country (Martikainen and Sakki, 2021). This study addresses a gap in research on the COVID-19 pandemic and nationalism, and an absence of published empiric evidence of nationalism in the New Zealand media during COVID-19.

## METHODS

### Search Strategy and Data Collection

We sought data in English language print and online news media that were related to COVID-19 from the ProQuest database that accesses 19 New Zealand newspapers. We downloaded all ProQuest news articles written in English and published from January 2020 to June 2022 that contained at least one of the following terms: coronavirus OR corona virus OR COVID-19 OR COVID OR COVID-19 OR pandemic.

A simple random sample of all articles that met search criteria was drawn from all articles across the 19 newspapers, stratified by 6-month time period. The time periods were 1 January 2020–30 June 2020, 1 July 2020–31 December 2020, 1 January 2021–30 June 2021, 1 July 2021–31 December 2021 and 1 January 2022–30 June 2022.

Sample size was driven by the qualitative analysis described below and determined using the principle of saturation (Vasileiou et al., 2018). Saturation was assessed among 500 articles in the period 1 January 2020–30 June 2020, and achieved with 200 articles per period for subsequent periods. For quantitative analyses, 200 articles per period with *α* = 0.05 yielded two-sided power of 0.9 to detect changes between periods in the prevalence of themes from 0 to 0.05, 0.48 to detect changes from 0.05 to 0.10 and 0.8 for changes from 0.10 to 0.20.

### Data Analysis

Our research followed the steps in thematic analysis outlined by Braun and Clarke (2006). After becoming familiar with the dataset (Step one), we developed the codebook (Step two) using emergent inductive coding (Hsieh and Shannon, 2005).

The data were read, key text segments identified and labeled with a code and reviewed. Two authors, EMRA and EF, inductively, concurrently, and independently read and coded a random sample of articles from time period one (*n* = 50), then compared, reviewed and refined the codebook categories and coding processes. EMRA and EF then simultaneously and independently tested the codebook with a new random subset of articles from time period two (*n* = 50). EMRA and EF coded a third random subset from time period five (*n* = 50) with the revised codebook. The final codebook and coding instructions were used to code the remaining articles across all sample sets by EMRA. We coded at all levels: sentence, paragraph and key phrase, to capture the multiple meanings in an article (Chandra and Shang, 2019). The intercoder reliability of coding in the periods 1 January–30 June 2020 and 1 January–30 June 2022 was assessed as 86 per cent and 93 per cent respectively. NVivo20 (Lumivero, Colorado, United States) was used to manage content coding.

The statistical frequency of codes, confidence intervals and statistical significance of differences were determined. The chi-squared test was used to assess differences in code prevalences between time periods. The null hypothesis was that there was no significant difference in the prevalence of codes across time periods.

### Development of Themes

Following coding, the codes were grouped into themes to help to answer and used the guiding theory of nationalism (Billig, 1995; Braun and Clarke, 2006). The themes were developed in a two-step iterative process of development and reflection in combination with the qualitative analysis and quantitative statistical analysis (Step 3). The two-step process of theme development involved assessing quantitative data, such as the frequency of codes, that highlighted potential themes. Other potential themes emerged from language, rhetorical power and tonal qualities coded for in the qualitative analysis. The data associated with each theme were read, and themes were considered in the context of the entire dataset to ensure themes were coherent and distinct from each other (Step 4). We reported prevalent codes and codes notable to theme development. Extracts from media articles were selected to illustrate themes, and these are presented after quantitative results on themes and codes. These extracts were attributed to the journalist unless stated otherwise.

### Quantitative Analysis of Themes

Once themes emerged, all articles were classified based on whether they contained each identified theme. The prevalence of each theme emerging from the qualitative analysis among articles by time period was expressed with 95% confidence intervals (Table 2). Changes in the prevalence of these themes between periods were compared with chi-squared tests for trend across ordinal time periods. The null hypothesis was that there was no significant difference in the prevalence of themes across time periods.

**Table 2.** Trends in themes of domestication, unification, separation and securitization in New Zealand print media by time period, January 2020 to June 2022

Theme	All articles (*n* = 1300) *n* (%, 95% CI^a^)	Most common codes per theme (*n* = 1300) *n* (%, 95% CI)	Time Period 1 Jan–Jun 2020 (*n* = 500) *n* (%, 95% CI)	Time Period 2 Jul–Dec 2020 (*n* = 200) *n* (%, 95% CI)	Time Period 3 Jan–Jun 2021 (*n* = 200) *n* (%, 95% CI)	Time Period 4 Jul–Dec 2021 (*n* = 200) *n* (%, 95% CI)	Time Period 5 Jan–Jun 2022 (*n* = 200) *n* (%, 95% CI)	*P*-value
Domestication	954 (73.4%, 95% CI [69.4, 77.3%])	New Zealand economy: 543 (41.7%, 95% CI [30.0, 44.5%]) New Zealand COVID-19 notifications: 212 (16.3%, 95% CI [14.3, 18.4%])	390 (78.0%, 95% CI [74.2, 81.7%])	123 (61.5%, 95% CI [57.2, 65.9%])	160 (80.0%, 95% CI [76.4, 83.6%])	134 (67%, 95% CI [62.8, 71.2%])	114 (57.0%, 95% CI [52.6, 61.4%])	<0.001
Unification	778 (59.9%, 95% CI [55.5, 64.2%])	Sport: 248 (19.1%, 95% CI [16.9, 21.3%]) Unifying language: 140 (10.8%, 95%CI [9.1, 12.4%])	428 (85.6%, 95% CI [82.5, 88.7%])	88 (44.0%. 95% CI [39.6, 48.4%])	101 (50.5% 95% CI [46.0, 55.0%])	53 (26.5%, 95% CI 22.6, 30.5%)	16 (54.0%, 95% CI [49.5, 58.5%])	<0.001
Securitization	293 (22.5%, 95% CI [18.8, 26.3%])	Borders: 142 (10.9%, 95% CI [9.1, 12.7%]) War language: 102 (7.9% (95% CI [6.4, 9.3%])	93 (18.6%, 95% CI [15.1, 22.1%])	55 (27.5%, 95% CI [23.5, 31.5%])	47 (23.5%, 95% CI [19.7, 29.3%])	47 (23.5%, 95% CI [19.7, 29.3%])	51 (25.5%, 95% CI [21.6, 29.4%])	0.075
Separation	234 (18.0%, 95% CI [14.6, 21.4%])	Migrants: 66 (5.1%, 95% CI [3.9, 6.3%]) Māori and Pasifika people: 54 (4.2%, 95% CI [3.0, 5.3%])	67 (13.4%, 95% CI [10.4, 16.5%])	29 (14.5%, 95% CI [11.4, 17.7%])	37 (18.5%, 95% CI [15.0, 22.0%])	41 (20.5%, 95% CI [16.9, 24.1%])	60 (30.0%, 95% CI [25.9, 34.1%])	<0.001

^a^95% Confidence Interval.

## Results

Our search yielded 48,696 articles published from January 2020 to June 2022 (Table 1). Based on thematic saturation, a total of 1300 articles were coded; 500 in January through June 2020, and 200 for each of the remaining time periods.

**Table 1.** Article yield from the initial search result on the ProQuest database, and the subsequent number of articles analyzed for coding

	Total from January 2020 to June 2022	January 2020–June 2020	July 2020–December 2020	January 2021–June 2021	July 2021–December 2021	January 2022–June 2022
Article yield from search, duplicates removed	25,756	5604	6307	4061	5738	4036
Articles analyzed	1300	500	200	200	200	200

Codes included topics (e.g. sport, the economy), language (e.g. pronouns like ‘we’), and tonal qualities (e.g. New Zealand portrayed in a positive, negative or neutral light when compared to another country). A total of 77 codes were identified (Supplementary Material).

Following the coding process, we found thematic convergence in the reporting about COVID-19 in New Zealand around four themes of nationalistic sentiment in New Zealand media: domestication, unification, securitization and separation. These four themes structure the next section of the results.

### Domestication

The theme of domestication involved the representation of COVID-19 through a predominately New Zealand lens. Of 1300 articles, 954 (73.4 per cent, 95% CI, 69.4, 77.3 per cent) contained the theme of domestication with a significant downward trend in prevalence of the theme over time (Table 2). The most common codes for this theme were the New Zealand economy and New Zealand COVID-19 notification numbers (Table 2). Of the 1300 articles 51 (3.9 per cent, 95% CI [2.9, 5.0 per cent]) were coded for the global impact of COVID-19; 16 (1.1 per cent, 95% CI [0.6, 1.8 per cent]) were coded for the impact of COVID-19 on a specific country other than New Zealand; 11 (0.9 per cent, 95% CI [0, 1.7 per cent]) reported on the global distribution of vaccines; 110 (8.5 per cent, 95% CI [6.9, 10.1 per cent]) reported the total global COVID-19 notifications; 6 (0.5 per cent, 95% CI [0.1, 1.9 per cent]) reported the COVID-19 notifications of a specific country such as Australia, Italy, or the United States and 41 (3.2 per cent, 95% CI [2.2, 4.1 per cent]) reported notified SARS-CoV-2 infections that had been imported into New Zealand from another country.^1^

#### Global and local news

Global COVID-19 notifications tended to be reported as an aggregate global statistic only alongside the New Zealand COVID-19 notifications in ‘*At A Glance*’ articles outlining the daily notifications from the New Zealand government of COVID-19 illnesses and deaths.

In our sample news articles emphasized how information from other countries was not regarded with the same importance as information from New Zealand:

Extract 1: ‘Anytime something like this happens, like a virus or an outbreak, there are always two sides. I take the information from New Zealand, not overseas, and the risk in New Zealand is very minimal to no risk’ [quote from an interviewed parent] (Blommerde, 2020).

Domestication is underscored in the expression to ‘shop local’ to grow the nation’s economy during COVID-19:

Extract 3: And yet again, 15 or 20 per cent of what you pay goes to the big guy offshore. It’s hard for someone who believes in the free-market economy to write some of this stuff…Buy local. Book it direct. And let’s see if we can help one another out of this mess (Cotterill, 2020).

### Unification

The theme unification involved the homogenous unification of New Zealand, through the rhetoric of New Zealand self-image, and the use of pronouns like ‘we’ and ‘us’. Of 1300 articles, 778 (59.9 per cent, 95% CI [55.5, 64.2 per cent]) included the theme of unification with a significant downward trend in prevalence of the theme over time (Table 2). The most common codes for this theme were sports and unifying language (Table 2). In addition, of the 1300 articles that compared New Zealand to another country 24 (1.9 per cent, 95% CI [1.1, 2.6 per cent]) were coded as neutral, 20 (2.3 per cent 95% CI [1.5, 3.2 per cent]) were coded as New Zealand compared in a negative light, and 86 (6.6 per cent, 95% CI [5.2, 8.0 per cent]) were coded as New Zealand compared in a positive light.

In our sample, news articles quoted ‘us’, ‘we’, or ‘our’ language used by officials to encourage adherence, evidenced in the extracts below:

Extract 3: Together us and the community will be able to unite against COVID-19 and put New Zealand in the best chance for recovery. [quoting All of Government Controller, John Ombler] (Dubby, 2020).

Extract 4: Our commitment is to help each other as we work through the crisis that our country faces. We’re all in this together [quoting Auckland mayor Phil Goff] (Auckland Council, 2020).

In these examples, ‘us’, ‘our’ and ‘we’ centered persons as members of the nation-state of New Zealand as their primary form of identity. It is worth noting that ‘we’ and ‘our’ were not always used to denote the entire nation. For example, in this statement from the former New Zealand Minister of Health, Dr David Clark, ‘our’ refers to the shared commitment of the government:

Extract 5: these measures are important to protect the health of New Zealanders, which is our number one priority [quoting Minster of Health, Dr David Clark] (Auckland Council, 2020).

#### Sports

Other articles emphasized the importance of sport in unifying the nation. For example, at the introduction of a sport recovery package from the government it was reported that:

Extract 6: sport plays a significant role in bringing our communities together. Our success on the world stage also brings inspiration and pride to our country (New Zealand Government, 2020).

#### New Zealand image

The extract below emphasizes how news articles portrayed New Zealand in a positive light:

Extract 7: As the pandemic continues to reverberate around the world, New Zealand is standing tall on the global stage in terms of clear and decisive action, communication and humanity (Truslove, 2020).

### Securitization

The theme of securitization involved the establishment of strict border entry requirements and the consideration of the COVID-19 virus as a security threat. Of 1300 articles, 293 (22.5 per cent, 95% CI [18.8, 26.3 per cent]) included the theme of securitization with no significant change in the prevalence of the theme over time (Table 2). The most common codes for this theme were borders and war language (Table 2).

#### Borders

Our sampled articles discussed managed isolation and quarantine facilities at the border, border testing, border workers and conflict over whether the borders should reopen. For example, Extract 8 conveyed the primary reason to maintain closed borders was to keep COVID-19 out of New Zealand, and as COVID-19 cases rose in New Zealand, the border was no longer the primary defense:

Extract 8: COVID-19 modeller Michael Plank said the border opening would not make much difference to the number of cases, which soared from 15,000 before the Omicron outbreak to more than three-quarters of a million. ‘We have had very high rates of Covid here in New Zealand... So, adding in some border cases is not going to have a huge impact.’ (Stuff reporters, 2022).

Despite expert reassurance on reopening the border, Extract 9 reports the public sentiment towards opening the borders, and borders are still seen as the primary method for controlling COVID-19:

Extract 9: It will be no small challenge to change the now entrenched mindset of many of the New Zealand public. Even now, with very high levels of vaccinations, a sizeable clump of people think MIQ should stay in place and stricter measures be taken to thwart Omicron (The New Zealand Herald, 2022a).

#### War language

The following extracts show the language of war used by political leaders, including Prime Minister Jacinda Ardern, and public health scientists:

Extract 10: ‘in this fight against the virus, we have some things on our side… from midnight tonight we bunker down… as we fight to save New Zealander’s lives... our number of cases may be small, but that doesn’t mean we have yet been successful in hunting the virus down.’(New Zealand Parliament Hansard, 2020)

Extract 11: Public health expert and epidemiologist Professor Michael Baker says complacency is also the enemy when trying to eliminate the virus (Parahi, 2020).

Other articles described a ‘fight against misinformation’ (Kenny, 2020). Border and healthcare workers were described as the ‘frontline’ (3.5 per cent, 95% CI [1.9, 5.1 per cent]). The cost-of-living crisis and inequities were described as ‘collateral damage’ (The New Zealand Herald, 2022b). ‘D-day’ was the name given to the end of lockdowns (Wanganui Chronicle, 2020), Government assistance was the ‘war chest’ (Cooke, 2020), and going to the supermarket was considered ‘sending out onto enemy line’ (Parahi, 2020). On the arrival of the vaccine in New Zealand, the vaccine became the ‘weapon’ and ‘armor’ against COVID-19 (New Zealand Government, 2021).

### Separation

The theme of separation involved the separation of nonhomogenous groups using language like ‘they’ and ‘them’. Of the 1300 articles, 234 (18.0 per cent, 95% CI [14.6, 21.4 per cent]) contained the themes of separation, with a significant upwards trend in prevalence of the theme over time (Table 2). The most common codes for this theme were migrants and Māori and Pasifika (Table 2).

#### Migrants, immigrants and emigrants

In our sample articles reported on the need to instead bring expatriate New Zealanders back to New Zealand, as well as negative views on migrants:

Extract 13: New Zealand’s reliance on foreign migrant labour, and in some industries its exploitation of that labour, has been highlighted by the closed borders’ [quoting the president of Council of Trade Unions] (Carroll, 2021).

Extract 14: The Deputy Prime Minister yesterday said the Government told foreigners at the start of the COVID-19 crisis that if their circumstances had changed dramatically, they should go home (Wade, 2021).

#### Māori and Pasifika people

In our sample, articles discussed Māori and Pasifika people often in relation to achieving equity, for example:

Extract 12: In New Zealand, the choice is also about equity and fairness. Māori and Pacific people are roughly twice as likely to suffer cardiovascular disease and diabetes, and up to five times as likely to suffer from respiratory conditions (Hogan, 2020).

One article expressed an opposing view:

Extract 9: In fact the only groups to openly flout the lockdown rules and seek ongoing publicity for their law-breaking were tribal activists running illegal blockades to promote their separatist agenda (Newman, 2020).

#### Tourists

As COVID-19 emerged in New Zealand in early 2020, and at the news of new variants of SARS-CoV-2 circulating around the world such as the Delta variant in 2021 (Ministry of Health, 2022), articles in our sample noted the threat tourists posed to New Zealanders:

Extract 17: One would hope in cases where tourists arrogantly ignore the restrictions, the law is rigorously applied, and such people are deported immediately [comment from a member of public] (Beck, 2020).

As with migration, an over-reliance on tourism was also questioned as unsustainable with borders closed:

Extract 18: Our community is very much based on the tourism economy. Many in our community, and I count myself as one of those, look to a future where we are no longer so dependent on tourism. We must diversify our economy (The Daily Post, 2020).

Chinese tourists were singled out and reliance on Chinese tourism questioned, demonstrated below from February 2020:

Extract 19: Perhaps it’s a timely opportunity to review our increasing reliance on Chinese visitation and the quality of the visitor experience we provide (Yardley, 2020).

When the New Zealand borders were announced to reopen in May 2022, the conversation turned to welcoming international tourists and their contribution to the national and local economy. Journalists were also aware of the sentiment towards others:

Extract 21: However, New Zealanders have had two long years of believing travellers will bring the evil Covid in. That has not changed — many will be worried (The New Zealand Herald, 2022a).

#### Countries other than New Zealand

Articles reported both cooperative and competitive narratives towards other countries. For example, quoting an American news website headline: ‘The pandemic winner: Will It be Sweden or New Zealand?’ (Waikato Times, 2020).

In contrast:

Extract 22: As the world emerges from the COVID-19 pandemic, the message of Laudato Si’, that all of life is interconnected, is a timely reminder of the need for coordinated action to overcome interrelated environmental, economic and social crises (Caritas Aotearoa, 2020).

## Discussion

We identified nationalist themes of domestication, unification, securitization and separation that were common in New Zealand media during the COVID-19 pandemic. The themes of domestication, unification, securitization, and separation were most prominent in the period from January through June 2020, suggesting nationalist rhetoric in the print media was strongest during the early stages of the pandemic when there was heightened fear and uncertainty, border closures and a need for solidarity. Domestication was evident in the number of news articles that reported on the domestic impact of COVID-19 compared to the global impacts or impacts in countries other than New Zealand. Based on our analysis of New Zealand media coverage of the COVID-19 pandemic, the nation remained a powerful image and central point of reference for unifying and invoking the group identity of the implied readers. There was also separation of groups such as migrants or tourists. The tension between the health of all humans and the health of New Zealanders was highlighted in the rhetoric of competition and comparison between New Zealand and the rest of the world. Nationalist themes were further realized in the securitization of COVID-19, with war language highlighting this tension, particularly in the first time period January–June 2020.

Domestication of New Zealand news enabled the formation of a comparison and competition-based identity. While it is not surprising that local newspapers would report on local news, COVID-19 was global phenomenon rather than a local or national one (Berger, 2009). Domestication gives news coverage a distinctly New Zealand voice, aiding the advancement of a New Zealand self-image and concepts of conformity (Bishop and Jaworski, 2003). As Billig’s work on banal nationalism shows, the use of pronouns ‘our’ and ‘we’ enables the reader to imagine themselves as full members and party to a common national cause (Billig, 1995). Domestication unifies the country under a symbolic framework of identity and the competition was to remain the best compared to other countries on a ‘global stage’. Competition with countries other than New Zealand was emphasized over cooperation and was embedded in New Zealand self-image and rhetoric through comparison and sports, of which sports are considered a milder form of war (Watson, 2017; Arnold, 2021; Sturm et al., 2021). War service and sports are both considered key components of New Zealand national identity, suggesting competition is embedded in the national image (Crump et al., 2023). The reporting of global and New Zealand COVID-19 notifications considered New Zealand as one group and the rest of the world as the other. Competitive identity formation and groupings suggest COVID-19 notifications could be interpreted as a running scorecard of performance (Crump et al., 2023). Furthermore, Skilling found that New Zealand identity was largely structured by global norms such as flexibility and innovation (Skilling, 2010). Being flexible and innovative therefore, is something that New Zealand may use as a point of outperformance to other countries, rather than something that is contributed to the global stage.

Securitization of disease threats is a strategic formulation of nationalism. With borders the primary method of protection against COVID-19 in New Zealand, images of war rhetoric were used to portray COVID-19 as a security threat (Yang, 2022). The dichotomy between ‘human health’ at a global level and the physical, social, and economic health of New Zealand was highlighted. Further, there was a tension between protecting those within borders and extending cooperation beyond the nation-state. The use of conflict manifesting through military metaphors aligns with other research on the securitization of COVID-19 (Castro Seixas, 2021). Considering the global proliferation of war metaphors during COVID-19, securitization of health, nationalism and the resulting competition is not unique to the New Zealand context (Brencio, 2020). Despite intentions of uniting against a common viral enemy, such rhetoric links health with militarism and division, pitting groups against one another rather than encouraging collaboration (Varma, 2020). Our research provides empirical evidence that New Zealand’s COVID-19 media response was infused with nationalism that portrayed the pandemic response as a global competition that New Zealand was winning. The themes identified in this media analysis taken together advance a competitive rather than a cooperative global health narrative.

When surveyed at a community level, research suggests that New Zealanders perceive themselves as generous and willing to contribute to global equity (Kolandai et al., 2024). As a nation, New Zealand also claims to be founded upon principles of egalitarianism (Jacobs and Malpas, 2018). However, a gap exists between proposed actions and actual behavior. For instance, analyses of New Zealand philanthropy suggest that individual charity tends to align with the theme of domestication. When a sample of New Zealanders was asked to choose between donating to a national charity and a comparable global charity, 70.9 per cent of respondents opted for the domestic charity (Genç et al., 2021). Additionally, a social media analysis of New Zealanders living abroad during COVID-19 revealed polarized debates on who qualified as a ‘true New Zealander’, highlighting the dynamic between the themes of securitization, unification, and separation (Oden, 2022). Evidence suggests that nationalist messaging was not only present in the media but also resonated with segments of the population, reinforcing national identity in response to a global crisis (Gilray, 2021)

At the governmental level, it has been argued that New Zealand has ‘fall[en] victim to various forms of social myopia, nationalist boosterism, and sub-cultural narcissism’ (Jacobs and Malpas, 2018: 543). While the New Zealand COVID-19 response was successful in terms of illness and deaths averted, this was achieved through solidarity at a national level, rather than by appealing to global solidarity. While our results do not prescribe specific policy directions, they help explain why calls for global solidarity (Taylor et al., 2020), cooperation, and multilateralism (Bieber, 2022; Crump et al., 2023) often struggle to gain traction despite their ethical appeal. The global distribution of COVID-19 vaccines, and New Zealand’s contribution to the vaccine distribution network COVID-19 Vaccines Global Access (COVAX), the vaccines pillar of the Access to COVID-19 Tools (ACT) Accelerator provides a useful example. Like many other wealthy countries, New Zealand purchased more vaccines in advance than were needed to supply its population, fell short of its pledged contribution to COVAX, and donated vaccines to nations in the Pacific region, a region of geopolitical self-interest to New Zealand, only after it had decided not to use them for its own population (Powles, 2018; Crump et al., 2023). Moreover, while New Zealand has been vocal in its commitment to global development, it remains one of the least generous Organization for Economic Co-operation and Development countries in terms of bilateral and multilateral aid, particularly in health (Crump et al., 2023). Government policy, therefore, aligns with the theme of domestication and appears to reflect broad, although not universal public attitudes and behavior. Thus, while cooperation is a recurring theme in public and policy rhetoric, our findings, alongside other research, indicate that competition is likely to translate into concrete action and policy.

In contrast, any rhetoric of cooperation often remains aspirational rather than operationalized, suggesting a disconnect between stated values and actions. Generosity appears to be domestically anchored, and broad in principle yet shallow in practice. Both public attitudes and policy reinforce domestic prioritization, which limits meaningful contributions to global solidarity. When governments or populations adopt a nationalist orientation, they create tensions in recognizing robust obligations to nonnationals and may even display moral ambivalence toward those beyond state borders (Huysseune, 2009).

In the context of an infectious disease pandemic, a nationalist orientation may generate positive progress on measures to control disease threats domestically, such as disease surveillance, nonpharmaceutical interventions and vaccine development. However, global control of current pandemics, as well as long-term investment in the prevention of future global outbreaks, may be neglected (Wenham, 2019). The success of collaborations like COVAX depends on a willingness to address the needs of nonnationals alongside those of co-nationals, even without the promise of short-term benefit to contributing state. That is, COVAX requires a global health ethic underpinned by principles of moral cosmopolitanism which help operationalize the equal moral worth of all individuals. Pragmatically, in a global public health emergency, the well-being of all is dependent on a robust cooperative response, giving all states at least a self-interested reason to act to benefit those outside their borders.. However, vast global health inequities, both during and beyond pandemics, justify accepting the challenge of a cosmopolitan orientation. Cosmopolitanism is a plausible alternative to nationalism as a moral orientation for global health, one that emphasizes multilateralism and global cooperation, includes state and nonstate parties, and prioritizes the health of all people everywhere over the interests of individual states in order to promote justice and equity in health (Ruger, 2020; Jecker et al., 2022).

The World Health Organization is currently coordinating negotiation of a global ‘pandemic treaty’, which may propel some states to adopt a global health ethic underpinned by cosmopolitan principles. However, critics argue that the pandemic treaty in its current form will not overcome the nationalist tendencies of states nor contribute to more global health cooperation (Wenham et al., 2022; Anderson *et al.*, 2025). Furthermore, history shows that once the crisis period of a public health emergency is over the conditions that contributed to it often remain unchanged (Fenton and Chillag, 2021). Nationalist policies, such as vaccine stockpiling and export restrictions on essential supplies (Islam, 2022), while effective at protecting those within borders, contributed to the inequitable global distribution of essential supplies. These inequities in turn contributed to the prolonging of the pandemic as well as violating the human right to health (Murhula and Singh, 2022).

### Limitations

Our study provides empirical evidence of nationalism in New Zealand media during the COVID-19 pandemic that should be considered when developing global and public health policy. However, as discussed above, media is just one lens through which to examine the existence and strength of nationalism as reflected in public and political dialogue. Media analyses should be complemented by additional empirical evidence, such as the International Social Survey Programme (Kolandai et al., 2024).

In this analysis, we studied articles in newspapers with local and regional coverage within New Zealand. Such newspapers could be reasonably assumed to publish news that both reflects and influences their readership’s perceptions of COVID-19 policy. However, not all New Zealand newspapers were included in the ProQuest database and local papers may be expected to be weak on their coverage of global issues. Further, our study did not assess the importance of individual news stories or newspapers to its readership. While we were well placed to detect meaningful changes, our power to detect small changes in the prevalence of themes and codes between periods was limited. Future studies could extend our print media research to analyze public perception of the New Zealand response to COVID-19 and of global cooperation through analysis social media comments and posts, or through qualitative interviews.

## Conclusions

Nationalism can provide safety and security to readers of news media during a crisis like COVID-19, as readers can identify themselves in a perpetuated collective narrative. However, through domestication, unification, securitization, and separation, nationalism simultaneously promotes in-group solidarity while creating boundaries against ‘others’, in turn risking that nations separate and compete with nonnational groupings. Media analysis of reporting during the COVID-19 pandemic provides insight into the perpetuation of competitiveness during an event in which global cooperation is crucial. Our findings align with those of others highlighting the complex relationship between the COVID-19 pandemic and nationalism (Nossem, 2020; Ozkirimli, 2020; Rachman, 2020; Elias et al., 2021; Martikainen and Sakki, 2021; Bieber, 2022; Jenne, 2022; Crump et al., 2023). New Zealand has been considered by some as exemplifying a more positive form of nationalism and has been praised globally for its successful COVID-19 response (Jenne, 2022). However, our study found that the New Zealand media response was infused with themes of nationalism. On the one hand, this helped to drive a COVID-19 response that was successful in terms of illnesses and deaths averted. On the other, it tended to marginalize principles of cosmopolitanism that underpin a successful, equitable global response. Globally, tensions between these frameworks resulted in inequitable vaccine distribution and uncoordinated policy responses and ultimately prolonged the pandemic’s global impact. Yet nationalism’s domestic benefits illustrate why more cosmopolitan approaches face practical challenges despite their ethical appeal. We recommend that political leaders and public health experts avoid exclusively appealing to nationalist sentiments to build national solidarity, instead appealing to global solidarity, and prioritizing those in greatest need.

## Supplementary Material

phaf009_suppl_Supplementary_Material

## Footnotes

1 All codes are included in the Supplementary Material.
