# Supplementary material for: Nationalism in New Zealand Media During the COVID-19 Pandemic: A Mixed Methods Study
Source: Public Health Ethics. 2025 Sep 5;18(3):phaf009. doi: 10.1093/phe/phaf009 (PMC12410985; doi:10.1093/phe/phaf009)
Supplement: phaf009_suppl_Supplementary_Material [file phaf009_suppl_supplementary_material.docx]

Supplementary Material
*Frequencies, Percentages and 95% Confidence Intervals for All Codes Across Time Periods*

| Codes | Jan-Jun 2020 | % | Lower 95% CI | Upper 95% CI | Jul-Dec 2020 | % | Lower 95% CI2 | Upper 95% CI2 | Jan-Jun 2021 | % | Lower 95% CI3 | Upper 95% CI3 | Jul-Dec 2021 | % | Lower 95% CI4 | Upper 95% CI4 | Jan-Jun 2022 | % | Lower 95% CI5 | Upper 95% CI5 | Total | Total% | Lower 95% CI6 | Upper 95%CI |
| --- | --- | --- | --- | --- | --- | --- | --- | --- | --- | --- | --- | --- | --- | --- | --- | --- | --- | --- | --- | --- | --- | --- | --- | --- |
| Alert levels or traffic light system | 51 | 10.20% | 7.49% | 12.91% | 9 | 4.50% | 1.57% | 7.43% | 25 | 12.50% | 7.82% | 17.18% | 23 | 11.50% | 6.99% | 16.01% | 17 | 8.50% | 4.56% | 12.44% | 125 | 9.31% | 7.98% | 11.25% |
| Borders | 25 | 5.00% | 3.05% | 6.95% | 37 | 18.50% | 13.01% | 23.99% | 22 | 11.00% | 6.58% | 15.42% | 28 | 14.00% | 9.09% | 18.91% | 30 | 15.00% | 9.95% | 20.05% | 142 | 10.92% | 9.19% | 12.65% |
| Transtasman, pacific bubble | 6 | 1.20% | 0.23% | 2.17% | 7 | 3.50% | 0.90% | 6.10% | 15 | 7.50% | 3.78% | 11.22% | 8 | 4.00% | 1.23% | 6.77% | 7 | 3.50% | 0.90% | 6.10% | 43 | 3.15% | 2.32% | 4.30% |
| Case numbers |  |  |  |  |  |  |  |  |  |  |  |  |  |  |  |  |  |  |  |  |  |  |  |  |
| Global cases | 28 | 5.60% | 3.54% | 7.66% | 12 | 6.00% | 2.64% | 9.36% | 26 | 13.00% | 8.24% | 17.76% | 19 | 9.50% | 5.35% | 13.65% | 25 | 12.50% | 7.82% | 17.18% | 110 | 8.46% | 6.92% | 10.01% |
| Specific country case (other than NZ) | 2 | 0.40% | -0.16% | 0.96% | 0 | 0.00% | 0.00% | 0.00% | 1 | 0.50% | -0.50% | 1.50% | 3 | 1.50% | -0.22% | 3.22% | 0 | 0.00% | 0.00% | 0.00% | 6 | 0.46% | 0.09% | 0.84% |
| New Zealand cases | 79 | 15.80% | 12.54% | 19.06% | 22 | 11.00% | 6.58% | 15.42% | 34 | 17.00% | 11.69% | 22.31% | 36 | 18.00% | 12.57% | 23.43% | 41 | 20.50% | 14.79% | 26.21% | 212 | 16.31% | 14.26% | 18.36% |
| Imported country case | 21 | 4.20% | 2.41% | 5.99% | 6 | 3.00% | 0.59% | 5.41% | 5 | 2.50% | 0.29% | 4.71% | 3 | 1.50% | -0.22% | 3.22% | 6 | 3.00% | 0.59% | 5.41% | 41 | 3.15% | 2.18% | 4.12% |
| Domestic impact of covid |  |  |  |  |  |  |  |  |  |  |  |  |  |  |  |  |  |  |  |  |  |  |  |  |
| Cost of living | 1 | 0.20% | -0.20% | 0.60% | 5 | 2.50% | 0.29% | 4.71% | 7 | 3.50% | 0.90% | 6.10% | 4 | 2.00% | 0.02% | 3.98% | 8 | 4.00% | 1.23% | 6.77% | 25 | 1.92% | 1.16% | 2.68% |
| Crime or family violence | 5 | 1.00% | 0.11% | 1.89% | 5 | 2.50% | 0.29% | 4.71% | 4 | 2.00% | 0.02% | 3.98% | 1 | 0.50% | -0.50% | 1.50% | 3 | 1.50% | -0.22% | 3.22% | 18 | 1.38% | 0.74% | 2.03% |
| Economy (all) | 215 | 43.00% | 28.77% | 57.23% | 69 | 34.50% | 15.18% | 53.82% | 93 | 46.50% | 23.14% | 69.86% | 80 | 40.00% | 19.46% | 60.54% | 77 | 38.50% | 18.33% | 58.67% | 534 | 41.77% | 39.03% | 44.50% |
| Economy general | 49 | 9.80% | 7.14% | 12.46% | 15 | 7.50% | 3.78% | 11.22% | 17 | 8.50% | 4.56% | 12.44% | 25 | 12.50% | 7.82% | 17.18% | 17 | 8.50% | 4.56% | 12.44% | 123 | 9.46% | 7.84% | 11.09% |
| Business | 66 | 13.20% | 10.17% | 16.23% | 15 | 7.50% | 3.78% | 11.22% | 23 | 11.50% | 6.99% | 16.01% | 16 | 8.00% | 4.16% | 11.84% | 21 | 10.50% | 6.16% | 14.84% | 141 | 10.85% | 9.12% | 12.57% |
| Economic nationalism | 20 | 4.00% | 2.25% | 5.75% | 10 | 5.00% | 1.92% | 8.08% | 12 | 6.00% | 2.64% | 9.36% | 8 | 4.00% | 1.23% | 6.77% | 6 | 3.00% | 0.59% | 5.41% | 56 | 4.31% | 3.18% | 5.43% |
| Economy v health | 16 | 3.20% | 1.63% | 4.77% | 0 | 0.00% | 0.00% | 0.00% | 1 | 0.50% | -0.50% | 1.50% | 0 | 0.00% | 0.00% | 0.00% | 0 | 0.00% | 0.00% | 0.00% | 17 | 1.31% | 0.68% | 1.94% |
| Employment | 38 | 7.60% | 5.23% | 9.97% | 10 | 5.00% | 1.92% | 8.08% | 13 | 6.50% | 3.01% | 9.99% | 15 | 7.50% | 3.78% | 11.22% | 16 | 8.00% | 4.16% | 11.84% | 92 | 7.08% | 5.65% | 8.50% |
| Housing | 14 | 2.80% | 1.32% | 4.28% | 15 | 7.50% | 3.78% | 11.22% | 16 | 8.00% | 4.16% | 11.84% | 9 | 4.50% | 1.57% | 7.43% | 11 | 5.50% | 2.28% | 8.72% | 65 | 5.00% | 3.79% | 6.21% |
| Supply chain | 12 | 2.40% | 1.03% | 3.77% | 4 | 2.00% | 0.02% | 3.98% | 11 | 5.50% | 2.28% | 8.72% | 7 | 3.50% | 0.90% | 6.10% | 6 | 3.00% | 0.59% | 5.41% | 40 | 3.08% | 2.12% | 4.03% |
| Education | 20 | 4.00% | 2.25% | 5.75% | 7 | 3.50% | 0.90% | 6.10% | 4 | 2.00% | 0.02% | 3.98% | 10 | 5.00% | 1.92% | 8.08% | 10 | 4.00% | 1.23% | 6.77% | 51 | 3.92% | 2.85% | 5.00% |
| Entertainment | 3 | 0.60% | -0.09% | 1.29% | 3 | 1.50% | -0.22% | 3.22% | 4 | 2.00% | 0.02% | 3.98% | 5 | 2.50% | 0.29% | 4.71% | 4 | 2.00% | 0.02% | 3.98% | 19 | 1.46% | 0.80% | 2.13% |
| Events | 12 | 2.40% | 1.03% | 3.77% | 11 | 5.50% | 2.28% | 8.72% | 14 | 7.00% | 3.39% | 10.61% | 13 | 6.50% | 3.01% | 9.99% | 12 | 6.00% | 2.64% | 9.36% | 62 | 4.77% | 3.59% | 5.95% |
| Health sector | 42 | 8.40% | 5.92% | 10.88% | 4 | 2.00% | 0.02% | 3.98% | 10 | 5.00% | 1.92% | 8.08% | 7 | 3.50% | 0.90% | 6.10% | 21 | 10.50% | 6.16% | 14.84% | 84 | 6.46% | 5.10% | 7.83% |
| PPE | 3 | 0.60% | -0.09% | 1.29% | 2 | 1.00% | -0.41% | 2.41% | 3 | 1.50% | -0.22% | 3.22% | 0 | 0.00% | 0.00% | 0.00% | 0 | 0.00% | 0.00% | 0.00% | 8 | 0.62% | 0.18% | 1.05% |
| Mental health | 11 | 2.20% | 0.89% | 3.51% | 5 | 2.50% | 0.29% | 4.71% | 5 | 2.50% | 0.29% | 4.71% | 3 | 1.50% | -0.22% | 3.22% | 8 | 4.00% | 1.23% | 6.77% | 32 | 2.46% | 1.60% | 3.32% |
| Tourism | 19 | 3.80% | 2.09% | 5.51% | 12 | 6.00% | 2.64% | 9.36% | 12 | 6.00% | 2.64% | 9.36% | 9 | 4.50% | 1.57% | 7.43% | 12 | 6.00% | 2.64% | 9.36% | 64 | 4.92% | 3.72% | 6.12% |
| Transport | 11 | 2.20% | 0.89% | 3.51% | 0 | 0.00% | 0.00% | 0.00% | 2 | 1.00% | -0.41% | 2.41% | 5 | 2.50% | 0.29% | 4.71% | 2 | 1.00% | -0.41% | 2.41% | 20 | 1.54% | 0.86% | 2.22% |
| Environment | 19 | 3.80% | 2.09% | 5.51% | 15 | 7.50% | 3.78% | 11.22% | 11 | 5.50% | 2.28% | 8.72% | 9 | 4.50% | 1.57% | 7.43% | 11 | 5.50% | 2.28% | 8.72% | 65 | 5.00% | 3.79% | 6.21% |
| Ethics, rights, equity | 11 | 2.20% | 0.89% | 3.51% | 4 | 2.00% | 0.02% | 3.98% | 9 | 4.50% | 1.57% | 7.43% | 10 | 5.00% | 1.92% | 8.08% | 18 | 9.00% | 4.95% | 13.05% | 52 | 4.00% | 2.91% | 5.09% |
| Global impact of covid | 23 | 4.60% | 2.73% | 6.47% | 4 | 2.00% | 0.02% | 3.98% | 11 | 5.50% | 2.28% | 8.72% | 6 | 3.00% | 1.80% | 10.80% | 7 | 3.50% | 0.90% | 6.10% | 51 | 3.92% | 2.85% | 5.00% |
| Groups |  |  |  |  |  |  |  |  |  |  |  |  |  |  |  |  |  |  |  |  |  |  |  |  |
| Extremist, far right groups | 0 | 0.00% | 0.00% | 0.00% | 1 | 0.50% | -0.50% | 1.50% | 0 | 0.00% | 0.00% | 0.00% | 0 | 0.00% | 0.00% | 0.00% | 7 | 3.50% | 0.90% | 6.10% | 8 | 0.62% | 0.18% | 1.05% |
| Global solidarity | 11 | 2.20% | 0.89% | 3.51% | 1 | 0.50% | -0.50% | 1.50% | 1 | 0.50% | -0.50% | 1.50% | 2 | 1.00% | -0.41% | 2.41% | 1 | 0.50% | -0.50% | 1.50% | 16 | 1.23% | 0.62% | 1.84% |
| Kiwis overseas | 8 | 1.60% | 0.48% | 2.72% | 9 | 4.50% | 1.57% | 7.43% | 5 | 2.50% | 0.29% | 4.71% | 5 | 2.50% | 0.29% | 4.71% | 7 | 3.50% | 0.90% | 6.10% | 34 | 2.62% | 1.73% | 3.50% |
| Māori and Pacific | 14 | 2.80% | 1.32% | 4.28% | 5 | 2.50% | 0.29% | 4.71% | 9 | 4.50% | 1.57% | 7.43% | 12 | 6.00% | 2.64% | 9.36% | 14 | 7.00% | 3.39% | 10.61% | 54 | 4.15% | 3.05% | 5.26% |
| Migrant | 21 | 4.20% | 2.41% | 5.99% | 12 | 6.00% | 2.64% | 9.36% | 15 | 7.50% | 3.78% | 11.22% | 7 | 3.50% | 0.90% | 6.10% | 11 | 5.50% | 2.28% | 8.72% | 66 | 5.08% | 3.86% | 6.29% |
| Other countries | 6 | 1.20% | 0.23% | 2.17% | 1 | 0.50% | -0.50% | 1.50% | 2 | 1.00% | -0.50% | 2.41% | 3 | 1.50% | -0.22% | 3.22% | 5 | 2.50% | 0.29% | 4.71% | 17 | 1.31% | 0.68% | 1.94% |
| Tourists | 7 | 1.40% | 0.35% | 2.45% | 0 | 0.00% | 0.00% | 0.00% | 3 | 1.50% | -0.22% | 3.22% | 4 | 2.00% | 0.02% | 3.98% | 5 | 2.50% | 0.29% | 4.71% | 19 | 1.46% | 0.80% | 2.13% |
| Unvaccinated | 0 | 0.00% | 0.00% | 0.00% | 0 | 0.00% | 0.00% | 0.00% | 2 | 1.00% | -0.41% | 2.41% | 8 | 4.00% | 1.23% | 6.77% | 10 | 5.00% | 1.92% | 8.08% | 20 | 1.54% | 0.86% | 2.22% |
| Unifying language | 79 | 15.80% | 12.54% | 19.06% | 19 | 9.50% | 5.35% | 13.65% | 13 | 6.50% | 3.01% | 9.99% | 13 | 6.50% | 3.01% | 9.99% | 16 | 8.00% | 4.16% | 11.84% | 140 | 10.77% | 9.05% | 12.49% |
| Be kind | 16 | 3.20% | 1.63% | 4.77% | 3 | 1.50% | -0.22% | 3.22% | 0 | 0.00% | 0.00% | 0.00% | 0 | 0.00% | 0.00% | 0.00% | 1 | 0.50% | -0.50% | 1.50% | 20 | 1.54% | 0.86% | 2.22% |
| Winners v losers | 3 | 0.60% | -0.09% | 1.29% | 0 | 0.00% | 0.00% | 0.00% | 2 | 1.00% | -0.41% | 2.41% | 0 | 0.00% | 0.00% | 0.00% | 1 | 0.50% | -0.50% | 1.50% | 6 | 0.46% | 0.09% | 0.84% |
| Impact of COVID-19 on specific country other than NZ | 6 | 1.20% | 0.23% | 2.17% | 0 | 0.00% | 0.00% | 0.00% | 0 | 0.00% | -0.50% | 1.50% | 1 | 0.50% | -0.50% | 1.50% | 9 | 4.50% | 1.57% | 7.43% | 16 | 1.23% | 0.62% | 1.84% |
| Informative | 25 | 5.00% | 3.05% | 6.95% | 0 | 0.00% | 0.00% | 0.00% | 4 | 2.00% | 0.02% | 3.98% | 9 | 4.50% | 1.57% | 7.43% | 11 | 5.50% | 2.28% | 8.72% | 49 | 3.77% | 2.71% | 4.83% |
| International guidelines or treaties | 12 | 2.40% | 1.03% | 3.77% | 5 | 2.50% | 0.29% | 4.71% | 2 | 1.00% | -0.41% | 2.41% | 2 | 1.00% | -0.41% | 2.41% | 3 | 1.50% | -0.22% | 3.22% | 24 | 1.85% | 1.10% | 2.59% |
| Local council | 1 | 0.20% | -0.20% | 0.60% | 1 | 0.50% | -0.50% | 1.50% | 6 | 3.00% | 0.59% | 5.41% | 1 | 0.50% | -0.50% | 1.50% | 1 | 0.50% | -0.50% | 1.50% | 10 | 0.77% | 0.28% | 1.25% |
| Lockdown violations | 13 | 2.60% | 1.18% | 4.02% | 0 | 0.00% | 0.00% | 0.00% | 4 | 2.00% | 0.02% | 3.98% | 7 | 3.50% | 0.90% | 6.10% | 4 | 2.00% | 0.02% | 3.98% | 28 | 2.15% | 1.35% | 2.96% |
| New Zealand compared to other country |  |  |  |  |  |  |  |  |  |  |  |  |  |  |  |  |  |  |  |  |  |  |  |  |
| Neutral | 13 | 2.60% | 1.18% | 4.02% | 1 | 0.50% | -0.50% | 1.50% | 4 | 2.00% | 0.02% | 3.98% | 1 | 0.50% | -0.50% | 1.50% | 5 | 2.50% | 0.29% | 4.71% | 24 | 1.85% | 1.10% | 2.59% |
| NZ in negative light | 7 | 1.40% | 0.35% | 2.45% | 3 | 1.50% | -0.22% | 3.22% | 9 | 4.50% | 1.57% | 7.43% | 3 | 1.50% | -0.22% | 3.22% | 8 | 4.00% | 1.23% | 6.77% | 30 | 2.31% | 1.47% | 3.16% |
| NZ in positive light | 50 | 10.00% | 7.32% | 12.68% | 11 | 5.50% | 2.28% | 8.72% | 14 | 7.00% | 3.39% | 10.61% | 4 | 2.00% | 0.02% | 3.98% | 7 | 3.50% | 0.90% | 6.10% | 86 | 6.62% | 5.24% | 7.99% |
| New Zealand, nation, context | 43 | 8.60% | 6.09% | 11.11% | 6 | 3.00% | 0.59% | 5.41% | 5 | 2.50% | 0.29% | 4.71% | 3 | 1.50% | -0.22% | 3.22% | 9 | 4.50% | 1.57% | 7.43% | 66 | 5.08% | 3.86% | 6.29% |
| NZ image | 18 | 3.60% | 1.93% | 5.27% | 5 | 2.50% | 0.29% | 4.71% | 5 | 2.50% | 0.29% | 4.71% | 4 | 2.00% | 0.02% | 3.98% | 8 | 4.00% | 1.23% | 6.77% | 40 | 3.08% | 2.12% | 4.03% |
| NZ in international media | 2 | 0.40% | -0.16% | 0.96% | 2 | 1.00% | -0.41% | 2.41% | 0 | 0.00% | 0.00% | 0.00% | 0 | 0.00% | 0.00% | 0.00% | 5 | 2.50% | 0.29% | 4.71% | 9 | 0.69% | 0.23% | 1.15% |
| Politics |  |  |  |  |  |  |  |  |  |  |  |  |  |  |  |  |  |  |  |  |  |  |  |  |
| Election | 13 | 2.60% | 1.18% | 4.02% | 17 | 8.50% | 4.56% | 12.44% | 0 | 0.00% | 0.00% | 0.00% | 0 | 0.00% | 0.00% | 0.00% | 5 | 2.50% | 0.29% | 4.71% | 35 | 2.69% | 1.79% | 3.59% |
| Foreign affairs | 4 | 0.80% | 0.00% | 1.60% | 3 | 1.50% | -0.22% | 3.22% | 0 | 0.00% | 0.00% | 0.00% | 5 | 2.50% | 0.29% | 4.71% | 3 | 1.50% | -0.22% | 3.22% | 15 | 1.15% | 0.56% | 1.75% |
| Leadership | 66 | 13.20% | 10.17% | 16.23% | 23 | 11.50% | 6.99% | 16.01% | 13 | 6.50% | 3.01% | 9.99% | 11 | 5.50% | 2.28% | 8.72% | 23 | 11.50% | 6.99% | 16.01% | 136 | 10.46% | 8.76% | 12.16% |
| Opposition | 14 | 2.80% | 1.32% | 4.28% | 12 | 6.00% | 2.64% | 9.36% | 5 | 2.50% | 0.29% | 4.71% | 4 | 2.00% | 0.02% | 3.98% | 13 | 6.50% | 3.01% | 9.99% | 48 | 3.69% | 2.65% | 4.74% |
| Policy | 18 | 3.60% | 1.93% | 5.27% | 1 | 0.50% | -0.50% | 1.50% | 7 | 3.50% | 0.90% | 6.10% | 8 | 4.00% | 1.23% | 6.77% | 13 | 6.50% | 3.01% | 9.99% | 47 | 3.62% | 2.58% | 4.65% |
| Government assistance | 42 | 8.40% | 5.92% | 10.88% | 2 | 1.00% | -0.41% | 2.41% | 2 | 1.00% | -0.41% | 2.41% | 8 | 4.00% | 1.23% | 6.77% | 2 | 1.00% | -0.41% | 2.41% | 56 | 4.31% | 3.18% | 5.43% |
| Public health measures | 7 | 1.40% | 0.35% | 2.45% | 2 | 1.00% | -0.41% | 2.41% | 3 | 1.50% | -0.22% | 3.22% | 4 | 2.00% | 0.02% | 3.98% | 9 | 4.50% | 1.57% | 7.43% | 25 | 1.92% | 1.16% | 2.68% |
| Bubble' | 2 | 0.40% | -0.16% | 0.96% | 17 | 8.50% | 4.56% | 12.44% | 4 | 2.00% | 0.02% | 3.98% | 1 | 0.50% | -0.50% | 1.50% | 2 | 1.00% | -0.41% | 2.41% | 26 | 2.00% | 1.22% | 2.78% |
| Contact tracing, testing | 6 | 1.20% | 0.23% | 2.17% | 5 | 2.50% | 0.29% | 4.71% | 10 | 5.00% | 1.92% | 8.08% | 7 | 3.50% | 0.90% | 6.10% | 12 | 6.00% | 2.64% | 9.36% | 40 | 3.08% | 2.12% | 4.03% |
| Masks | 5 | 1.00% | 0.11% | 1.89% | 11 | 5.50% | 2.28% | 8.72% | 5 | 2.50% | 0.29% | 4.71% | 1 | 0.50% | -0.50% | 1.50% | 6 | 3.00% | 0.59% | 5.41% | 28 | 2.15% | 1.35% | 2.96% |
| Recovery | 24 | 4.80% | 2.89% | 6.71% | 21 | 10.50% | 6.16% | 14.84% | 28 | 14.00% | 9.09% | 18.91% | 15 | 7.50% | 3.78% | 11.22% | 16 | 8.00% | 4.16% | 11.84% | 104 | 8.00% | 6.50% | 9.50% |
| New normal | 6 | 1.20% | 0.23% | 2.17% | 2 | 1.00% | -0.41% | 2.41% | 2 | 1.00% | -0.41% | 2.41% | 3 | 1.50% | -0.22% | 3.22% | 4 | 2.00% | 0.02% | 3.98% | 17 | 1.31% | 0.68% | 1.94% |
| Science | 7 | 1.40% | 0.35% | 2.45% | 2 | 1.00% | -0.41% | 2.41% | 7 | 3.50% | 0.90% | 6.10% | 4 | 2.00% | 0.02% | 3.98% | 4 | 2.00% | 0.02% | 3.98% | 24 | 1.85% | 1.10% | 2.59% |
| Technology | 16 | 3.20% | 1.63% | 4.77% | 2 | 1.00% | -0.41% | 2.41% | 5 | 2.50% | 0.29% | 4.71% | 3 | 1.50% | -0.22% | 3.22% | 7 | 3.50% | 0.90% | 6.10% | 33 | 2.54% | 1.67% | 3.41% |
| Sports | 67 | 13.40% | 10.35% | 16.45% | 34 | 17.00% | 13.64% | 20.36% | 34 | 17.00% | 13.64% | 20.36% | 23 | 11.50% | 8.65% | 14.35% | 23 | 11.50% | 8.65% | 14.35% | 248 | 19.08% | 16.90% | 21.26% |
| Vaccine | 8 | 1.60% | 0.48% | 2.72% | 4 | 2.00% | 0.02% | 3.98% | 27 | 13.50% | 8.67% | 18.33% | 30 | 15.00% | 9.95% | 20.05% | 41 | 20.50% | 14.79% | 26.21% | 110 | 8.46% | 6.92% | 10.01% |
| Global distribution | 1 | 0.20% | -0.20% | 0.60% | 1 | 0.50% | -0.50% | 1.50% | 7 | 3.50% | 0.90% | 6.10% | 2 | 1.00% | -0.41% | 2.41% | 0 | 0.00% | 0.00% | 0.00% | 11 | 0.85% | 0.00% | 1.70% |
| Mandates and certificates | 0 | 0.00% | 0.00% | 0.00% | 0 | 0.00% | 0.00% | 0.00% | 0 | 0.00% | 0.00% | 0.00% | 11 | 5.50% | 2.28% | 8.72% | 10 | 5.00% | 1.92% | 8.08% | 21 | 1.62% | 0.92% | 2.31% |
| Misinformation or conspiracies | 0 | 0.00% | 0.00% | 0.00% | 0 | 0.00% | 0.00% | 0.00% | 5 | 2.50% | 0.29% | 4.71% | 5 | 2.50% | 0.29% | 4.71% | 3 | 1.50% | -0.22% | 3.22% | 13 | 1.00% | 0.45% | 1.55% |
| War language | 59 | 11.80% | 8.91% | 14.69% | 11 | 5.50% | 2.28% | 8.72% | 8 | 4.00% | 1.23% | 6.77% | 11 | 5.50% | 2.28% | 8.72% | 13 | 6.50% | 3.01% | 9.99% | 102 | 7.85% | 6.35% | 9.34% |
|  |  |  |  |  |  |  |  |  |  |  |  |  |  |  |  |  |  |  |  |  |  |  |  |  |
